# Embryo-Based Large Fragment Knock-in in Mammals: Why, How and What’s Next

**DOI:** 10.3390/genes11020140

**Published:** 2020-01-29

**Authors:** Steven Erwood, Bin Gu

**Affiliations:** 1 Program in in Genetics and Genome Biology, Hospital for Sick Children, Toronto, ON M5G 0A4, Canada; steven.erwood@sickkids.ca; 2 Department of Molecular Genetics, University of Toronto, Toronto, ON M5S 1A8, Canada; 3 Program in Developmental and Stem Cell Biology, Hospital for Sick Children, Toronto, ON M5G 0A4, Canada

**Keywords:** CRISPR-Cas9, genome editing, large fragment knock-in, HDR, embryo

## Abstract

Endonuclease-mediated genome editing technologies, most notably CRISPR/Cas9, have revolutionized animal genetics by allowing for precise genome editing directly through embryo manipulations. As endonuclease-mediated model generation became commonplace, large fragment knock-in remained one of the most challenging types of genetic modification. Due to their unique value in biological and biomedical research, however, a diverse range of technological innovations have been developed to achieve efficient large fragment knock-in in mammalian animal model generation, with a particular focus on mice. Here, we first discuss some examples that illustrate the importance of large fragment knock-in animal models and then detail a subset of the recent technological advancements that have allowed for efficient large fragment knock-in. Finally, we envision the future development of even larger fragment knock-ins performed in even larger animal models, the next step in expanding the potential of large fragment knock-in in animal models.

## 1. Introduction

**Figure 1:**
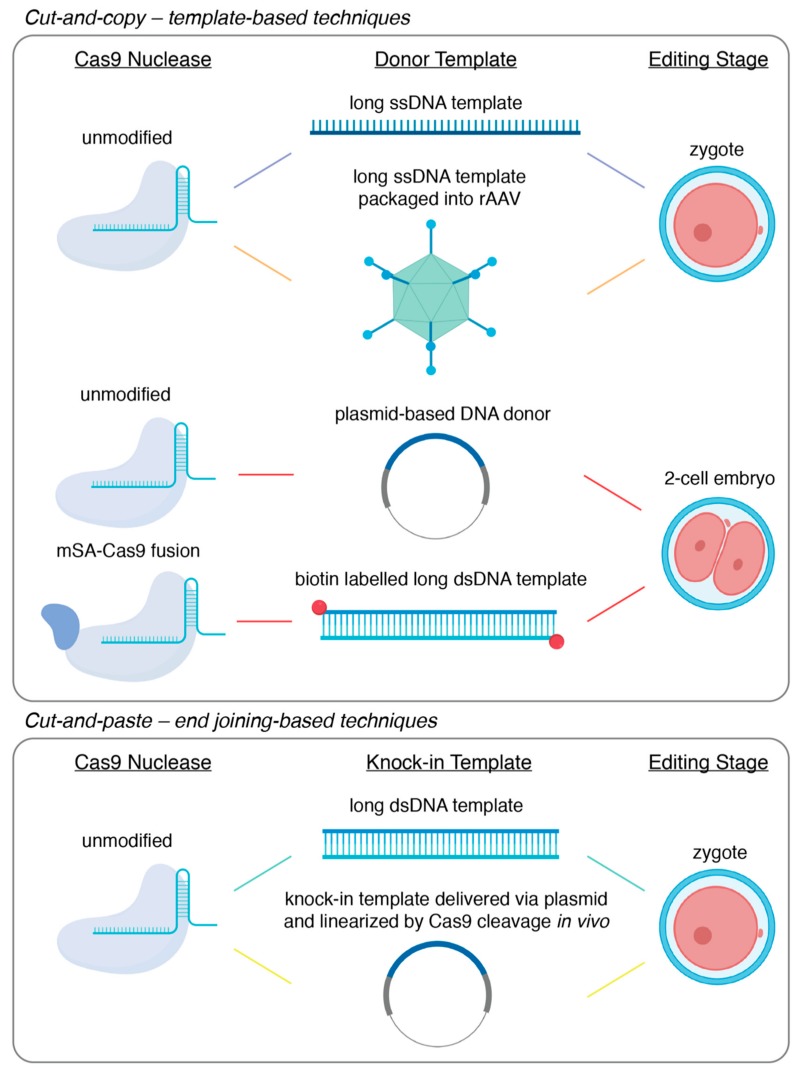
Visual summary of the technologies reviewed.

The development of endonuclease-mediated genome editing technologies, CRISPR/Cas9 in particular, has revolutionized animal genetics by opening up the possibility to achieve precise genome editing directly through embryo manipulations [1]. This has not only significantly improved the speed and efficiency of animal generation using typical mammalian model organisms such as mice and rats, but has also opened up the possibility to use traditionally harder-to-engineer species such as livestock and non-human primates for genetic research [2,3]. Of all the types of genetic modification that can be introduced through embryo manipulations, large fragment knock-in, which we practically define here as the seamless insertion or replacement of gene fragments of several hundreds to thousands of base pairs into an endogenous genomic locus, can allow for unique insights into both basic and translational research, ranging from developmental biology to disease modeling. From a technical point of view, however, large fragment knock-in remains one of the most challenging types of gene editing. In spite of this, many diverse techniques have been developed to achieve large fragment knock-in efficiently. In this review, we first discuss the main applications of large fragment knock-in in animal models, answering why knock-ins are uniquely important for the biological and biomedical sciences. Next, we discuss the recent progress and the technical improvements made for the efficient generation of large fragment knock-in. Finally, we present the current technical limits of large fragment knock-in and offer our view of how these may be surmounted to achieve the full benefits of large fragment knock-in in animal models. Here, it is worth noting that we aim to highlight only a subset of the important technological innovations representing the improvement of knock-in efficiency by distinct mechanisms (Figure 1), rather than to provide an exhaustive summary of the many useful techniques that define the field.

## 2. Why Large Fragment Knock-In?

### 2.1. Let There Be Light—Developmental Biology in Real-Time

Developmental biology seeks to understand the meticulously regulated process of how a single cell—a fertilized egg—can generate a highly complex yet organized organism. The most straightforward way to reveal the nature of these developmental processes is to follow them in real time using live imaging. While sought after for many years, watching development take place in real-time with molecular precision is now becoming routine as a result of recent technological developments on two fronts.

First, a large variety of fluorescent proteins, self-labeling tags, and luciferase tags have allowed for the live visualization of proteins by fluorescent and bioluminescent imaging within a broad optical spectrum—from blue to infra-red [4,5,6,7,8,9]. Moreover, bridging molecules, including catalytically inactive Cas9 proteins, MS2 loops, Sun-tags, and Moon-tags, have expanded the imageable molecular entities from proteins to include both DNA and RNA [10,11,12,13]. These advancements have created tremendous opportunities for visualizing each molecular entity of the central dogma in living embryos. Second, exciting new developments in imaging equipment have created unprecedented possibilities for long-term live imaging of embryonic development with high spatio (μm)-temporal (seconds to minutes) resolution. In particular, by applying advanced multiphoton microscopy [14] and light-sheet microscopy [15]—two unique physical mechanisms that both decrease the total photon exposure of a given sample and thus limit phototoxicity—critical processes including morphogenesis [16], cell lineage determination, and neural circuit establishment [17,18] have been traced in whole living embryos, including the often challenging gastrulating mouse embryos [19]. Equally important is the development of imaging analysis tools including the recent deep-learning-based methods [20]. These tools allow for the tracking of behavior, such as marker gene expression, signaling activation, and cell migration, of thousands of cells in a whole embryo over periods as long as several days. 

With these technological advancements, developmental biology is entering an exciting time of new discovery. There is, however, one gap remaining in the live imaging toolkit—embryos carrying multiple fluorescent reporters, recapitulating endogenous gene expression pattern and protein dynamics, which need to be obtained efficiently, either by direct embryo manipulation or breeding genetically modified animals. Traditionally, protein dynamics were investigated indirectly through the use of fluorescent proteins driven by partial regulatory sequences such as promoter or enhancer regions of a gene-of-interest [21]. These reporter constructs were integrated randomly into the genome. While this could theoretically be achieved by introducing transgene fragments to the embryos of any species, the technologies are better established in model organisms such as mice and zebrafish. Although this approach has been instrumental in revealing gene expression patterns in live embryos, random transgenesis has intrinsic drawbacks, including failing to recapitulate gene expression patterns due to the lack of full regulatory sequences, and expression variability between lines carrying the same transgene due to the local chromatin environment of the particular insertion site [22,23,24]. Moreover, recent systematic mapping of the insertion locus of a large number of transgenic mouse lines has revealed that the insertion of a transgene is often accompanied by complex genomic changes, including large insertions or deletions at the insertion site, which could lead to confounding phenotypes unrelated to the particular transgene [25]. Knock-in, defined as the seamless insertion of an exogenous sequence precisely into a genomic locus of design, could largely resolve these drawbacks and achieve faithful tagging of endogenous genes. Since the size of the coding sequences of most live imaging tags ranges from hundreds of base pairs to several kilo-base pairs, large fragment knock-in is required. Before the emergence of targeted endonuclease-assisted genome editing technologies, the generation of a knock-in reporter model organism was a difficult task, requiring years to complete. Even in mice, which are most amenable to large fragment knock-in due to the development of homologous recombination-based gene targeting technology through embryonic stem (ES) cells, the process of generating a knock-in by traditional means typically required screening of hundreds of ES cell clones, which was followed by time consuming chimera generation and breeding [26,27]. In other species, it is either impossible or entirely reliant on extremely inefficient processes such as somatic nuclear transfers (SCNT) [28]. New genome editing technologies, most notably the CRISPR/Cas9 system, have brought new hope for rapidly generating reporter embryos and animals through direct embryo manipulation for live imaging research in developmental biology, bypassing the typically tedious and time-consuming cell engineering steps. Although initial reports were promising, the efficiency of homologous recombination-mediated large fragment knock-in through mouse zygote injection tends to be variable and not robust [29,30]. Similarly, in other species, including frequently used model organisms such as zebrafish, the efficiency of large fragment knock-in remains low. To address this, many technological modifications, reviewed below, have been developed and applied to achieve highly efficient large fragment knock-in. To date, an impressively large number of knock-in reporters are being, or have been, generated using these methods. In our opinion, these robust technological innovations that allow for the highly efficient generation of knock-in reporters are bringing the exciting future of witnessing developmental biology live into sight. 

### 2.2. Understanding Our Past—Functional Evolution

As stated by Theodosius Dobzhansky, "*nothing in biology makes sense except in the light of evolution.*" Understanding how the changes in the DNA sequences of genomes, both coding and regulatory, shaped the stunning diversity of life forms is a fundamental question in biology. Due to the tremendous technological advancements that led to consistently decreasing costs of DNA sequencing, comparative genomics studies have revealed millions of sequence variants in both coding and regulatory sequences along the branches of the phylogenetic tree [31,32]. This has provided many promising candidate sequences, such that we can now ask fundamental questions of how the emergence of a new sequence variant might lead to phenotypes ranging from new developmental patterns, the emergence of new organs or even the origin of human specific functions such as speech [33,34]. 

A few examples eloquently support the idea that one critical way to answer these questions is to swap orthologous sequences between model organisms, which necessarily depends on large fragment knock-in technology. Likely shaped by environmental adaptation, the limb is one of the most phenotypically diverse structures in animals [35]. As is expected, many sequence variants have been discovered in enhancers close to the critical regulatory genes of limb development, such as sonic hedgehog (Shh) [36]. Using knock-in with CRISPR/Cas9 to formally prove the functional significance of these variants, Kvon et al. replaced the zone of polarization activity regulatory sequence (ZRS) enhancer, which controls the expression of Shh in animal limbs, in mice with the orthologous sequences from snakes and fish [36]. Indeed, the knock-in of the ZRS enhancer from snake species resulted in a radically truncated limb phenotype in mice, supporting that the ZRS sequence variants in snakes are likely responsible for the loss of limb structures in snakes throughout evolution. 

Another example pertains to a human-specific function; namely, the capacity for speech. Both human genetic disease and evolutionary genomics studies have implicated two human-specific amino acid variants in the transcription factor FOXP2 in the emergence of speech capacity in the human lineage [37]. Interestingly, when the mouse exon containing those two variants were replaced by knock-in with the human exon, the resultant genetically modified mouse presented modified basal ganglia circuits and ultrasonic vocalization. This provided experimental evidence for the involvement of the human-specific FOXP2 variants in human speech [38]. Of course, the functional roles of FOXP2 variants are far from clear, and a novel behaviour such as speech must have been shaped by the coordinated act of many genetic changes, the elucidation of which will require more discovery in genomics and subsequent functional testing in genetically humanized animals. 

Moreover, Sydney Brenner once proposed an interesting set of experiments to interrogate a fundamental question—between the coding sequences, which seem to be more functional, and the non-coding sequences, which actually comprise most of our genome, which of the two play a more essential role in shaping a functional organism? Brenner’s idea involves a unique fish, the pufferfish—*Takifugu rubripes* or fugu. This fish has a genome of roughly 1/8th the size of the human genome, and yet contains a similar number of coding genes [39,40]. Indeed, orthologs of most human coding genes can be found in the fish and they code for proteins comparable in size. Accordingly, these fish have significantly smaller non-coding genomes, with correspondingly smaller genic sequences that are very economically packaged and devoid of most so-called ‘junk sequences’. Sydney Brenner proposed that by systematically replacing mouse genes with their fugu orthologs, one could begin to understand the relative contribution of coding and non-coding sequence for each gene by phenotyping the ‘fugu-nized’ mice, and thus come closer to potential new understanding of whether or not the junk sequences are indeed ‘real junk’ on a gene-by-gene basis [41]. Unfortunately, this idea could not be fully realized at the time due to the in-efficient knock-in replacement technology, but now, with new genome editing technologies, this can be achieved with ease. 

On a slightly different but still related topic, large fragment swapping could also be applied to dissect the functional redundancy between homolog genes, which often generated by gene duplication. One contentious conjecture in evolutionary theory is that these homologs will often gain new functions because of loosened evolutionary constraints [42]. To support this hypothesis, however, a functional test is required to understand the differences between homologs. Moreover, an interesting question remains as to whether these homologs first diverged in the expression pattern and then function, or vice versa. A recent publication eloquently untangled this question for a set of Sox genes. By swapping the coding sequence of homolog Sox2 and Sox3 genes in mouse models, they demonstrated that the two homologs are functionally equivalent in the tissues where their expression overlaps, such as the testis and brain [43], suggesting that at least in the particular case of Sox2/Sox3 genes, expression divergence proceeded a functional divergence, and the homologs provide functional redundancy in certain overlapping tissues, at least in the restricted evolutionary time.

Thus, systematically interrogating genetic variants in evolution by knock-in replacements in mouse and other animal models could not only reveal the genetic mechanisms of critical events in evolution such as the morphogenic adaptation of limbs or the emergence of placenta or the neocortex, but could also help us to ‘throw out the junk’ of our genome and achieve a new understanding of the functional coordination of the coding and regulatory genome and functional redundancy between homolog genes. 

### 2.3. Mouse or Human – Genetic Humanization in Medical Research

The central goal of biomedical research is to understand and develop therapies for human disease. Due to their many advantages, including low housing cost, short reproductive cycle, and widely available inbred strains, rodents, and in particular mice, have long served as fundamental models for human disease as well as hosts for generating therapeutic reagents, such as monoclonal antibodies. The genetic difference between humans and mice, however, has posed several critical challenges in utilizing mouse models in biomedical research. We argue that genetically humanized mouse models, in which selected mouse genes are replaced by their human ortholog, have and will continue to play critical roles in advancing biomedical research. The genetic humanization of mouse models has gone through tremendous advancement in recent years, and we encourage readers to explore other excellent reviews for comprehensive overviews of genetically humanized mouse models [44]. Here, we use a few examples to illustrate the promises of genetically humanized mouse models in biomedical research.

One area for which genetically humanized mouse models hold great promise is in the field of infectious disease [45]. There are numerus infectious agents such as polio virus (PV), measles virus (MV), hepatitis B, C, and D viruses, human immunodeficiency virus (HIV), and Ebola virus that have a restricted host range of humans or higher primates [45]. The cost and ethical concerns surrounding using non-human primate models has posed profound challenges in studying these diseases. The host selectivity of these agents is often determined by the sequence, and in turn, the structural specificity of host factor proteins—most commonly a membrane receptor for virus attachment and entry into host cells. Excitingly, many human-specific host factors have been identified. Logically, one promising way to construct mouse models for human- or primate-specific infectious disease is to express the human factor in a mouse or replace the mouse ortholog with the human host factor. Indeed, random transgenic expression of human host factors has provided important insights and clinical advancements in research on pathogens including PV, MV and Hepatitis C virus (HCV) and Hepatitis D virus (HDV) [46]. However, as discussed above, random transgenesis can lead to many unexpected genetic lesions in animal models, amongst other drawbacks, and as such, knock-in replacement should eventually come to be the preferred method to generate genetically humanized mouse models. Indeed, it has recently been shown that with the partial humanization of the CD81 and OCLN genes, infection of Hepatitis C virus (HCV) could be reconstructed in mice, demonstrating the utility of knock-in replacement mouse models in infectious disease research [47]. For many infectious agents, however, multiple human specific factors are required for the establishment of the disease. For example, in HCV, the humanization of CD81 and OCLN genes in mice allowed for the entrance of the virus into cells, however viral replication failed [47]. Therefore, the systematic humanization of multiple factors will likely be required to achieve the promise of genetically humanized mice in infectious disease, which requires highly efficient and robust large fragment knock-in technologies.

It is now clear that genome editing technologies such as CRISPR/Cas9 have broad potential in treating human genetic disease by targeted correction of the disease-causing genetic variant or the manipulation of genetic modifiers. Pre-clinical studies in animal models are a critical step for a new therapy before clinical development. Since many of the critical features used to evaluate the efficacy and safety of a potential CRISPR/Cas9-based therapy, such as on-target correction efficiency and off-target mutation rates, are highly dependent on the particular target sequence, it is of the utmost importance to develop and evaluate new CRISPR/Cas9-based therapies using mouse models carrying the corresponding human disease-causing sequence alongside the surrounding sequences. A recent publication demonstrated the promises of this approach. Leber congenital amaurosis type 10 (LCA10) is an autosomal recessive condition caused by biallelic loss-of-function mutations in the *CEP290* gene [48]. The most common mutation is an intronic mutation – IVS26 – that creates a novel splice site, which results in the inclusion of a cryptic exon and a premature termination codon, and in turn mRNA degradation. A CRISPR/Cas9-based therapy named EDIT-101 was developed targeting this mutation [48]. By removing or inverting a DNA fragment surrounding the point mutation in patient-derived human cell lines, EDIT-101 was shown to efficiently correct the genetic defect and disease phenotypes. Importantly, EDIT-101 was then evaluated in vivo for its efficacy, delivery kinetics, and dosage effect in a previously established mouse model with regionally humanized sequences containing the disease-causing mutation [48]. The in vivo gene editing efficiency by EDIT-101 was then evaluated in wildtype non-human primates [48]. It is noteworthy that the humanized mouse model is the only step in this development process that was both in vivo—and thus recapitulating the challenges for a CRISPR/Cas9-based therapy in whole animals—and carrying the exact disease-causing mutation. This example eloquently demonstrates the central roles that genetically humanized mice could play in the development of CRISPR/Cas9-based therapies for human genetic disease. With the recent advancement in the technologies for generating large fragment replacement in mice, we and other researchers are now generating many new humanized mouse models for genetic disease. We expect these models to be instrumental in facilitating important advancements in both the understanding and treatment of inherited disease. 

In addition to the exciting applications of mouse models in the biomedical sciences, genome editing technologies could have broad potential in the development of improved livestock, which could contribute significantly to a solution to the food crisis we are facing with growing populations. We refer the readers to excellent reviews published recently on this matter [49]. Although many of the beneficial genetic variants are single nucleotide polymorphisms (SNPs) or small insert-deletion variants which could be introduced by non-homologous end joining (NHEJ) or single strand template repair (SSTR)-based methods, large fragment knock-in could prove beneficial when the introduction of a cluster of closely linked SNPs is the desired outcome. As discussed in the last section of this review, large fragment knock-ins in large animals are still technically challenging. We are confident, however, that with new technological developments, large fragment knock-in will be instrumental in the development of livestock with a broad range of beneficial traits.

## 3. How to Achieve Large Fragment Knock-In

Classical gene-targeting using embryonic stem cells has been quickly superseded by the use of programmable endonucleases, such as CRISPR/Cas9. Instead of relying on spontaneous low-frequency DNA damage at the site-of-interest, these endonuclease-based technologies allow for the introduction of the requisite double-stranded break (DSB) in a highly efficient and targeted manner. Furthermore, these technologies allow researchers to bypass embryonic stem cells and deliver the genome editing components via pronuclear injection into fertilized zygotes. Quickly following the demonstration of CRISPR/Cas9-mediated genome editing in mammalian cells, the generation of knock-out mice or knock-in mice carrying single nucleotide substitutions became routine [29,50,51]. The relative inefficiency of large fragment knock-in, however, necessitated the development of a wide variety of increasingly sophisticated methodologies. Here, we will focus on CRISPR/Cas9-based methods and divide the technical approaches to large fragment knock-in into cut-and-copy techniques, which rely on homology-directed repair templates, or cut-and-paste techniques, which incorporate knock-in fragments utilizing DNA end-joining. The techniques described here are summarized in Figure 1 and Table 1.

### 3.1. Cut-and-Copy—Template-Based Techniques

While unified by use of a DNA template and an occasionally clumsy acronym or initialism, the different template-based knock-in strategies are increasingly diverse. The first demonstration of a large fragment knock-in using CRISPR/Cas9 was performed by Yang and colleagues, where fluorescent tags were knocked-in on two endogenous loci, with efficiencies ranging from 10% to 20% [29]. This was achieved by microinjecting Cas9 mRNA, an sgRNA, and a plasmid-based homology template into the cytoplasm of newly fertilized zygotes. However, the efficiency of this particular technology has since been shown to be highly variable and dependent on genetic locus [30,59]. Since then, several technological modifications have been developed that have led to reported knock-in efficiencies of up to 95%–100% in some gene loci. 

Innovations in template-mediated knock-in strategies were, by necessity, focused on coaxing homology-directed repair (HDR) to occur. The first substantial improvements in HDR efficiency were a result of modifications of the homology template itself. In traditional gene targeting, double-stranded DNA in the format of a plasmid was typically used to for knock-in via homologous recombination [27,60]. It had been shown, however, that short *single*-stranded oligodeoxyribonucleotide (ssODN) templates served as better substrates for HDR and thus resulted in higher efficiency when knocking-in smaller fragments like single nucleotide polymorphisms or small gene-tags [29,50,61]. In a strategy called efficient additions with ssDNA inserts-CRISPR, or Easi-CRISPR, it was proposed that the efficiency of large-fragment insertion could be improved by the utilization of a long single-stranded DNA template [52,53]. In its original demonstration, knock-in via Easi-CRISPR was accomplished by the microinjection of ribonucleoprotein complexes of Cas9 protein bound with a complexed crRNA and tracrRNA guide alongside a long ssDNA template encoding the desired knock-in sequence flanked by 60 to 105 base homology arms. This approach was used to accomplish knock-in of fragments varying from 800 base pairs to 1.4 kilobase pairs with efficiencies ranging from 25% to 67% [53]. It is of note that, while gaining the maximum efficiency of Easi-CRISPR requires zygotic microinjection, it is in principle adaptable to electroporation though the delivery of the long ssDNA donor [52]. However, at present, long single-stranded DNA template synthesis remains a primary limitation of Easi-CRISPR, with the insertion of fragments greater than 1–2 kilobases remaining a challenge. Furthermore, it has been observed that the secondary structure that may be formed within a long single-stranded DNA molecule could lead to the skipping of donor sequences during repair [62]. This poses another challenge for precise knock-in using Easi-CRISPR.

A further technological advance—two-cell homologous recombination (2C-HR)-CRISPR—was provided by our group where insights from developmental biology were used to achieve consistent knock-in efficiencies greater than 10%, with some loci being targeted successfully with 50% efficiency [54]. It is well-established that HDR is predominantly active in the late S-G2 phases of the cell cycle [63]. Indeed, there have been numerous reports of how timing genome editing to coincide with this period in vitro led to significantly greater rates of editing events mediated by HDR [64,65]. We reasoned that the two-cell stage embryo, with its exceptionally long G2 phase that is relatively well-synchronized across embryos, might similarly permit higher rates of knock-in editing. Furthermore, it is noteworthy that zygotic genome activation, which is characterized by open-chromatin that may be more readily accessible to editing enzymes and donor DNA, occurs during the elongated G2 phase of two-cell embryos [66]. In a side-by-side comparison, the delivery of the editing components to a two-cell stage embryo resulted in a significant enhancement of knock-in efficiency compared to equivalent delivery into zygotes, increasing from 1%–5% to 30%–35% at the two loci tested. In its characterization, 2C-HR-CRISPR allowed for the knock-in of reporter alleles in up to 60% of funder mice, simply by changing the timing of delivery. Importantly, 2C-HR-CRISPR was shown to be highly efficient for relatively large knock-in size and was used to introduce fragments as large as 7.5 kilobases.

To further improve 2C-HR-CRISPR, we modified the system once more, incorporating template recruitment to the site of the DSB. It has been speculated that HDR is hindered by the search for homologous sequences following the introduction of a DSB [67]. It follows, then, that recruiting the donor template to the DSB site would increase rates of HDR. Template recruitment has been achieved in numerous ways, including the design of synthetic guide RNAs fused to ssDNA donors, covalent linkage of Cas9 RNPs to ssDNA donors, and delivery of Cas9-avidin fusion proteins alongside biotin-labelled donors [67,68,69,70]. Alongside ample evidence of the benefits of template recruitment from in vitro studies, preceding the publication of 2C-HR-CRISPR, the use of Cas9-avidin fusion mRNA co-delivered with a biotin-labelled ssDNA donor template was shown to permit knock-in at rates as high as 20% when delivered zygotically [67]. Template recruitment was incorporated into the 2C-HR-CRISPR system by co-delivering mRNA encoding a Cas9-monomeric streptavidin fusion (mSA), an sgRNA and a PCR-derived biotin labelled dsDNA donor template [54]. Upon the translation of the Cas9-mSA fusion protein, the mSA protein binds with high affinity to the biotin molecules appended to the donor template, thus recruiting it to the site of cleavage. With this modification, the 2C-HR-CRISPR system produced knock-in efficiencies of up to 95% in some genes at the founder mouse stage (e.g., *Gata6-Halo* reporter gene) and high germline transmission, with some targets reaching 100%.

While the techniques described above can reliably mediate large-fragment knock-in, in each case, maximum efficiency requires the direct microinjection of the genome editing components into zygotes or two-cell stage embryos. Accordingly, these techniques are limited by a high technical barrier to entry and are restricted in throughput and scalability. Since CRISPR/Cas9 components can be delivered with relative ease via electroporation, it is generally acknowledged that the delivery of long double- or single-stranded donor templates is the limiting step in simplifying the generation of knock-in mouse models. Multiple groups have addressed this by using adeno-associated viral vectors as a delivery vehicle for long DNA donors, bypassing the need for embryo microinjection [55,71,72]. The most recent strategy following this approach was CRISPR RNP electroporation and AAV donor infection, or CRISPR-READI [55]. Chen and colleagues paired their CRISPR-EZ electroporation delivery [73] of the CRISPR/Cas9 components with adeno-associated virus (AAV)-based delivery of donor DNA templates. Specifically, they found that the naturally occurring AAV1 serotype could transduce mouse zygotes with high efficiency and thus co-opted this viral vector as a delivery vector for otherwise difficult-to-deliver DNA donors. Accordingly, zygotes were pre-incubated for 6-hours to allow for AAV transduction and subsequent delivery of the donor DNA template and then electroporated with ribonucleoprotein complexes of Cas9 with an sgRNA. This technique achieved knock-in with efficiencies as high as 50% and was used to introduce fragments as large as 3.3 kilobases. Although CRISPR-READI greatly simplifies the technical generation of knock-in mouse models, it is limited by the 4.9 kilobase packaging capacity of AAV vectors.

### 3.2. Cut-and-Paste – End Joining-Based Techniques

The majority of template-based strategies were first developed in cell models before being transitioned into the generation of animal models. In spite of a wide variety of end joining-based techniques being well-established in cell models, however, the majority remain under-utilized or completely absent in the field of mammalian animal model generation [74,75,76]. Nonetheless, these techniques can offer a suitable alternative to template-based methodologies and have been demonstrably useful, though relatively infrequently used, in model generation.

While DSB repair is often divided into repair by HDR or NHEJ, the most prominent cut-and-paste methodologies utilize one of two lesser-known DNA repair pathways that can occur following a DSB—microhomology-mediated end joining (MMEJ) and homology-mediated end joining (HMEJ). In MMEJ or HMEJ, two resected DNA ends can be joined by homology, annealed and ligated, with MMEJ typically being defined by the homologous joining of regions shorter than 25–50 base pairs in length, and HMEJ typically being defined by the homologous joining of regions greater than 50 base pairs in length [56,77,78]. These repair pathways have been co-opted numerous times over in genome editing. 

The application of HMEJ in large-fragment knock-in mouse model generation is perhaps best exemplified by a technique called targeted integration of linearized dsDNA-CRISPR, or tild-CRISPR [56]. The development of tild-CRISPR followed significant evidence from in vitro work from cell models and iPSCs demonstrating that linear dsDNA templates with approximately 800 base pair homology arms are utilized by cellular machinery in HMEJ-based repair [79]. In tild-CRISPR, a linear double-stranded DNA donor template flanked with 800 base pairs of homology arms, called a tild donor, is used. Once designed and cloned into a circular vector, the tild donor can be linearized by PCR or restriction digest and delivered alongside Cas9 mRNA and an sgRNA via microinjection into zygotes. Upon delivery, it is proposed that the blunt ends of the genomic DSB are resected in tandem with the blunt-ends of the tild donor, revealing homologous regions which act as a substrate for HMEJ, seamlessly incorporating the tild donor into the DSB site [56]. In its original description, tild-CRISPR exhibited an average knock-in efficiency of 30% and was used to integrate fragments as large as 6 kilobases, although editing efficiency did decrease with size of insertion. Furthermore, the pre-linearization of the tild donor was demonstrated not to be strictly necessary for efficient knock-in in a study by Yao and colleagues, which preceded the publication of tild-CRISPR [78]. Yao and colleagues delivered a plasmid-based donor template consisting of the desired knock-in fragment flanked by 800 base pair homology arms with appended sequences for in vivo linearization by Cas9. When delivered alongside mRNA encoding Cas9 and a sgRNA, this donor plasmid facilitated knock-in rates as high as 26% in murine embryos. Of note, Yao and colleagues further demonstrated high knock-in efficiencies in the embryos of the monkey species *Macaca fasciularis*, though these experiments were evaluated at the blastocyst stage and not further [78].

While not prohibitive, the generation of a donor template with extensive homology arms is a limitation on many of the previously detailed editing approaches. This is minimized in MMEJ-based techniques in large fragment knock-in, which requires homology arms ranging from 20 to 40 base pairs in length. It has also been established that the MMEJ pathway is highly active in the repair of CRISPR/Cas9-mediated DSBs, with microhomology-based repair being evident in the majority of repair events in both human and mouse cells [29,80]. The benefits of MMEJ have been demonstrated in the adaptation of a technique called CRISPR/Cas9-based precise integration into the targeted chromosome, or CRIS-PITCh, which was first demonstrated in cell lines, silkworms and frogs [57], to the generation of large fragment knock-in models [58]. In CRIS-PITCh, a PITCh donor comprised of a plasmid containing the knock-in fragment flanked with 40 base pair homology arms is delivered alongside Cas9 RNPs to one-cell staged zygotes and linearized in vivo via guide sequences appended to each homology arm. Similar to tild-CRISPR and HMEJ, the genomic double-stranded break was proposed to be resected at the same time as the now linear PITCh-donor, after which the microhomologous regions are aligned and used for DNA repair. In its original demonstration for mouse model generation, the CRIS-PITCh system was used to generate a fluorescently tagged reporter mouse line and a mouse line with a floxed exon with efficiencies as high as 35% [58].

Although the historical precedent, which has been focused on template-based methods for large fragment knock-in, has likely played a role in current trends in animal model generation, the cut-and-paste techniques described above can provide unique advantages over the predominate cut-and-copy techniques. A variety of factors come into play when considering the generation of a large fragment knock-in animal model, including but not limited to molecular biology aptitude, desired founder frequency, technical skill in embryo manipulation, and transgene size. Fortunately, as described here, there is a wide diversity of approaches with undoubtedly more innovations currently under development.

### 3.3. Conditional Alleles—What is the Better Way

**Figure 2:**
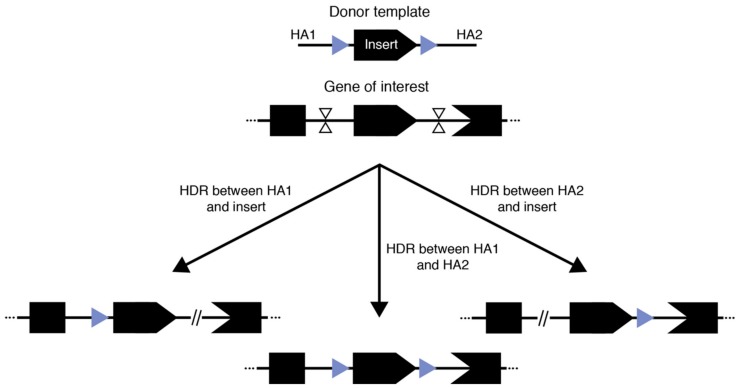
Three competing outcomes from generating floxed alleles with a large donor.

Mouse models bearing conditional alleles have had a profound impact on our understanding of functional genetics, especially in tissue- or temporal-specific contexts. The continued interest in the generation of conditional allele models spans across multiple disciplines. The traditional form of conditional alleles—floxed alleles—were generated by flanking a critical exon or multiple exons of an endogenous gene with loxP sequences in cis (Figure 2). Consequently, when a cre recombinase was expressed, typically in a tissue-specific or temporally controlled manner, the floxed sequence would be excised, leading to a frameshift mutation in the coding gene and thus the knock-out of that gene [81]. In theory, the two 34 bp loxP sites could be inserted independently by SSTR-mediated insertion using an ssODN. Although this method was originally reported to be efficient [29], a subsequent large-scale study proved that the efficiency of generating correct cis-located loxP sites flanking an exon is often problematic [50]. A technology for the sequential insertion of two loxP sites across two generations was recently reported as an effective means for floxed allele generation [82]. Although seemingly time consuming and laborious, with the established mouse in vitro fertilization technologies and newly developed ultra-superovulation methods [83,84], this may actually be a practical approach to the generation of floxed alleles, especially when the desired flanking region is long (e.g., several kbs or larger). 

Illustrated in the initial demonstrations of Easi-CRISPR and tild-CRISPR, another possible way to generate floxed alleles is to use long single- or double-stranded donor templates that contain the complete floxed region flanked by homology arms of varied length. In these approaches, two cuts are generated at the approximate position of the loxP sites and one of the cut-and-copy or cut-and-paste mechanisms discussed above are exploited to establish the insertion. Indeed, the principle underlying the success of these techniques has led to reported successful generation of floxed alleles with good efficiency outside of Easi- and tild-CRISPR [52,64,82]. However, there are two major limitations: (1) because the complete floxed allele needs to be contained in a plasmid, PCR product, or long single-stranded DNA, there is a clear size limit for the flanking region and (2) for methods reliant on HR-based incorporation using double-stranded DNA donors, our experience as well as communication with colleagues suggest that there is still a significant chance of having only a single loxP insertion. This is unsurprising since the region to be flanked by loxP sequences is homologous to the endogenous genome and therefore could also be recognized as a homology arm for the correction of one of the two double-stranded breaks, while the other break is repaired by NHEJ (Figure 2). Therefore, three outcomes are possible using this general approach: two outcomes where one break is corrected using HDR/HMEJ and the loxP flanked region as a homology arm while the other break is corrected using NHEJ, and one outcome where successful dual-loxP insertion occurs. Therefore, investigators have multiple factors to carefully consider when designing an experiment to generate floxed conditional alleles and should diligently genotype the resulting founders. 

Recently, a variety of degron systems that could mediate chemical-induced degradation of endogenously tagged proteins, such as the Auxin-inducible degradation system and the dTAG system, have been developed and have proven to be functional in mouse models [62,85,86]. These unique forms of conditional alleles provide a novel way to investigate gene function in model organisms. The various knock-in strategies described above are ideal for incorporating these systems, which are essentially large fragment fusion tags. We anticipate increased interest in the broad potential of these conditional alleles in future animal model generation, which will be assisted by all the large fragment knock-in technologies discussed in this review.

## 4. Future—Large and Larger

With all these new developments, we are on the edge of realizing new advancements in biomedical science using mouse models with large fragment knock-ins. However, we argue that there are still two technical challenges that once overcome will open up new grounds, both of which hinge on the word ‘larger’.

The first challenge is concerning ‘larger’ fragments. With almost all the knock-in technologies described up to now, the insert has to be contained in a plasmid or synthesized as a single-stranded DNA template. Accordingly, any insert that is greater than 10 kilobases could be challenging to achieve. However, many mammalian genes involved in genetic disease, particularly signaling receptors such as *FGFR2* (120 kilobases) and structural proteins such as dystrophin are significantly larger than 10 kilobases. Therefore, generating whole-gene humanized mouse models, which not only recapitulate the human coding sequence but also the regulatory sequences contained in introns and flanking untranslated regions, for studying these diseases requires much larger insertions. This logic holds true for studying evolution by whole gene replacement in mice as well. A potential way to overcome this challenge is to use artificial chromosomes that are capable of carrying hundreds of kilobases of DNA as repair donors [87]. One successful attempt was reported by Bergstrom and colleagues. By microinjecting bacterial artificial chromosome (BAC) donors into mouse zygotes, they achieved the humanization of a 25 kilobase segment of the human *Bcl2L11* gene [88]. While this is an exciting first step towards whole gene humanization in mice, the reported efficiency of 3.4% still needs improvement and a more thorough demonstration using more gene loci is required. We suggest that combining artificial chromosomes with the high homologous recombination activity during the G2 phase of two-cell stage mouse embryos could provide a promising route to achieve efficient whole gene humanization in mouse embryos. Further technology development should be directed here.

The second challenge concerns ‘larger’ animals. In spite of the many benefits of the mouse as a model organism, it is worth noting its many divergences from humans in terms of evolution, physiology, anatomy and development. It is clear that both developmental processes, such as the development of central nervous system (CNS), and human diseases, such as Huntington disease, cannot always be faithfully recapitulated using mouse models [89]. Knock-in disease models using large animals, such as the knock-in pig model for Huntington disease, have clearly demonstrated the significance of large animal models, particularly where mouse models are inadequate [90]. These knock-in models, however, are often generated by extremely inefficient and technically challenging technologies such as SCNT [90]. Accordingly, the clear utility of large animal models and non-human primates in biomedical research creates an urgent need for innovation in the development of molecular tools for the generation of models of this variety. Furthermore, since these larger animals tend to have longer reproduction times, the knock-in efficiency in one generation—and thus efficient manipulation of embryo genomes—is even more critical than in mice. We speculate that capitalizing upon a long G2 phase and active homologous recombination might be a generalized approach to achieving efficient large fragment knock-in in mammalian species. Although the cell cycle profile during early embryo development has not been clearly characterized in most species, it is logical to speculate that there might exist a long G2 phase corresponding to the zygotic genome activation in many mammalian species similar to what is found in mice. ZGA in mammals is the unique genome wide transcriptional activation of both single copy genic sequences and a broad range of repetitive sequences, which likely serves the purpose of opening up the dormant genome of the gametes [65]. If DNA replication proceeds at the same time of zygotic genome activation, there arises a conflict between the DNA replication and transcription machinery. The elongated G2 phase might serve as a compartmentalization mechanism for ZGA that avoids the replication–transcription conflict during early mammalian development. Of course, the validity of this argument needs to be tested by detailed cell cycle profiling during the early embryonic development across species. The cell cycle profiles gained through these experiments, however, would serve as a fundamental resource to develop long G2-based large fragment knock-ins to generate large animal models, and even, as has recently been proposed by Niakan and colleagues, to generate reporter human embryos to dissect early developmental events in our own species, under the appropriate ethical regulations, as summarized by Niakan’s recent review [91].

## Figures and Tables

**Table 1:** Summary of techniques reviewed.

Technique	Repair Mechanism	Characterizing Features	Primary Benefits
Easi-CRISPR [52,53]	HDR	Long single-stranded DNA donor with short homology arms	Achieves large fragment knock-in with efficiencies similar to small ssODN-based knock-ins
2C-HR-CRISPR [54]	HR	Microinjection into 2-cell staged embryo Template recruitment to the double-stranded break	Increased HR rates in 2-cell staged embryos lead to higher knock-in efficiency
CRISPR-READI [55]	HDR	Cas9 and gRNA electroporated into embryos followed by AAV-mediated delivery of donor template	Delivery of editing components does not require zygote microinjection
Tild-CRISPR [56]	HMEJ	Long, pre-linearized double-stranded donor with 800bp of homology	Linear double-stranded DNA donor overcomes size limitations of single-stranded DNA donors
CRIS-PITCh [57,58]	MMEJ	Plasmid-based donor with short homology arms linearized via Cas9 in vivo	Functions independently of HR, and requires minimal homology arm sequences
